# Muscular Fitness and Work Ability among Physical Therapists

**DOI:** 10.3390/ijerph18041722

**Published:** 2021-02-10

**Authors:** Yasmín Ezzatvar, Joaquín Calatayud, Lars Louis Andersen, Edgar Ramos Vieira, Rubén López-Bueno, José Casaña

**Affiliations:** 1 Exercise Intervention for Health Research Group (EXINH-RG), Department of Physiotherapy, University of Valencia, 46010 Valencia, Spain; yasmin.ezzatvar@uv.es (Y.E.); jose.casana@uv.es (J.C.); 2 National Research Centre for the Working Environment, 2100 Copenhagen, Denmark; LLA@nfa.dk (L.L.A.); rlopezbu@unizar.es (R.L.-B.); 3 Sport Sciences, Department of Health Science and Technology, Aalborg University, 9220 Aalborg, Denmark; 4 Department of Physical Therapy, Florida International University, Miami, FL 33199, USA; EVieira@fiu.edu; 5 Department of Physical Medicine and Nursing, University of Zaragoza, 50009 Zaragoza, Spain

**Keywords:** work ability index, physical performance, aging, physiotherapists, occupational health

## Abstract

The Work Ability Index (WAI) is a validated and widely used tool in occupational research. However, normative values for physical therapists (PTs) by age and sex are lacking. Although the nature of PTs’ work is physically demanding, it is unknown whether muscular fitness is associated with their WAI. This study sought to provide reference WAI data for Spanish PTs and to evaluate the association between PTs’ muscular fitness and WAI. Data on WAI of 1005 PTs were collected using a questionnaire. A subgroup (*n* = 68) performed a battery of physical tests including grip strength, push-ups and back-extension endurance. Associations between muscular fitness and WAI were evaluated using logistic regression controlling for various confounders. PTs aged 50 years or older had lower WAI scores than their younger counterparts. PTs with high back-extension endurance scored 3.5 (95% CI) higher in the WAI than those with low endurance. No associations were found between grip strength or number of push-ups and WAI. Our findings seem to highlight the importance of muscular fitness in PTs, especially the back-extension endurance.

## 1. Introduction

Work ability is defined as the balance between workers’ resources and the work demands [1]. Having good work ability is important for every worker during their whole working life [2]. Poor work ability is associated with early departure from the workforce [3], long-term sick leave, disability pension [4] and even mortality [5]. The Work Ability Index (WAI) is one of the most extensively used tools to estimate work ability and has been widely applied for both clinical and research purposes [1]. The index relies on different spheres of the worker’s life, including individual factors, the organizational structure of their job and the work environment, and it is highly dependent on age.

Aging workers are becoming a major concern for public health policies, mainly due to the exit of the workforce before the official retirement age [6], worsening overall health [7] and work ability decline [8]. In this context, while the mean age of the workforce is increasing, the physical demands of their jobs generally remain constant. Therefore, modifiable factors such as physical fitness will progressively become a cornerstone in helping workers remain in the workforce and improving their productivity, especially in physically demanding jobs.

Healthcare professionals represent a substantial part of the total workforce in industrialized countries. The physically demanding nature of their jobs could threaten their work ability. However, most research on healthcare professionals’ work ability has focused on nurses [9,10], and no WAI studies have been conducted on physical therapists (PTs). PTs are exposed to high physical demands, including sustained and awkward postures, bending, carrying, repositioning and lifting patients. Working postures and workload are associated with higher odds of having musculoskeletal pain [11]. Because musculoskeletal pain has a strong causal effect on work ability, the job physical demands could affect workers’ ability to work. Thus, improving workers’ physical fitness might be an efficient strategy to improve their work ability levels. Supporting this, previous studies have shown that performing high-intensity physical activity during leisure time has a dose–response relationship with work ability in workers with physically demanding jobs [12]; more specifically, performing vigorous physical activity more than 75 min per week was associated with lower levels of musculoskeletal pain among PTs [13]. Overall, work demands determine how great a role physical fitness has in the work ability of occupational groups. Upper body strength is deemed to be essential for performing PTs’ work-related tasks. However, strength generally declines as PTs’ age, negatively affecting their work ability.

While research has been carried out on the WAI in different occupational groups, no previous studies have investigated WAI values in PTs. This knowledge could help identify the most vulnerable PTs according to their age and sex and allow comparisons with other occupational groups. What is even less clear is the association of muscular fitness with WAI in PTs. Studying these associations is important for tailoring specific interventions aimed to improve their physical capacity and, therefore, to better physically prepare them to face their work-related tasks. Thus, the objectives of this study were to determine normative WAI values for Spanish PTs by age and sex and to investigate whether grip strength, push-ups and back-extension endurance are associated with WAI in PTs. We hypothesized that older PTs would have lower WAI than younger PTs and that those PTs with better muscular fitness levels will have higher work ability scores compared to those PTs with poorer muscular fitness.

## 2. Material and Methods

This cross-sectional study is part of a larger project investigating the working environment of Spanish PTs. Details of the procedures of the study have been reported elsewhere [13]. The present study received ethical approval from the University´s Ethical Committee (H1530736596718) in accordance with the principles of the Declaration of Helsinki and was designed and reported according to the Strengthening the Reporting of Observational Studies in Epidemiology (STROBE) guidelines to ensure comprehensive reporting of the data [14]. All data of the study were treated anonymously.

### 2.1. Questionnaire Content

The researchers contacted the main professional associations of PTs of different communities in Spain, who subsequently sent an e-mail invitation to their members inviting them to voluntarily participate. The invitation described the aim of the study, along with a link to the online questionnaire. The questionnaire was designed to collect information about work ability among PTs. Demographic questions regarding age, lifestyle factors and education were included to describe the nature and distribution of the sample. Due to data privacy reasons, the setting of the survey system was set to “anonymous”; i.e., it was not possible to link the individual responses to the individual emails of the participants.

#### Work Ability Assessment

Participants’ self-reported work ability was measured using the WAI questionnaire, including its seven subscales: (1) current work ability in comparison to lifetime best, (2) work ability in relation to the physical and mental demands of the job, (3) number of current diseases diagnosed by a physician, (4) estimated work impairment due to diseases, (5) sick leave during the past year, (6) own prognosis of work ability two years from now and (7) mental resources. The final score was calculated by summing up the estimated points for each item [15]. WAI score ranges from 7 to 49 points, distinguishing four different categories: poor WAI (7–27 points), moderate WAI (28–36), good WAI (37–43) and excellent WAI (44–49 points). The internal validity of this instrument has been previously described, finding a satisfactory relationship between subjective results of the index in comparison with more objective assessments [16,17], as well as a satisfactory test–retest reliability [18].

### 2.2. Muscular Fitness Assessment

The researchers contacted eligible hospitals and private clinics in the same area where the investigation was conducted. A subgroup of actively working PTs who had no job restrictions at the time of the examination and agreed to voluntarily participate were included in the study (n = 68). After completing the questionnaire, participants performed a battery of physical fitness tests including a hand-grip strength test, a back-extensor endurance test and push-ups. Tests were performed at their workplace in a single session, with the tests in a counterbalanced order across participants, in an attempt to minimize order effects. The test administrator was a PT with 3 years of experience in the application of physical fitness tests. Participants were asked to not ingest food, alcohol or caffeine or use tobacco products 3 h before the assessment. Moreover, subjects were instructed to be rested for the testing session, to sleep at least 7–8 h and to avoid significant exertion or exercise (more vigorous than usual daily activities) the day before the evaluation.

#### 2.2.1. Hand-Grip Strength

Hand-grip strength was measured using a TKK digital hand dynamometer (TKK 5101 Grip-D, Takey, Tokyo, Japan). The test administrator provided verbal instructions and a demonstration to the study participants regarding measurement procedures. Subjects were asked to maintain a standing position, with the shoulder adducted and neutrally rotated and arms parallel but not in contact with the body. After a familiarization test, three trials were performed with the dominant hand, encouraging participants to produce maximum force effort for 3 s. The highest value was recorded as the peak grip strength (kg) and was used for the analysis.

#### 2.2.2. Push-Ups

The push-up test was administered according to the American College of Sports Medicine (ACSM) protocol [19]. All participants started in the prone position. Male participants were asked to raise the body by straightening the elbows and return to the standard “down” position, and female subjects were instructed to perform the same exercise starting in the modified “knee push-up” position as described by the ACSM (with legs together, lower leg in contact with the floor, ankles plantar-flexed, back straight, hands shoulder-width apart, head up and knees as the pivotal point). The body was allowed to touch the floor after a push-up, but not rest on it. The test was stopped when the subject strained forcibly or was unable to maintain the appropriate technique within two repetitions. The final score was obtained by summing up the number of push-ups performed consecutively without rest.

#### 2.2.3. Back Muscular Endurance

The “Biering-Sørensen test” was used to assess the isometric endurance of back-extensor muscles. The test was performed according to previous recommendations [20]. High test–retest reliability has been found in a recent study for this test (ICC = 0.93–0.97) [21].

### 2.3. Statistical Analysis

All statistical analyses were performed using the SAS statistical software for Windows (Proc Logistic, SAS v9.4). Descriptive statistics were used to report demographic characteristics of the participants, including age, BMI, sex, education, amount of physical activity per week, smoking and alcohol consumption. Descriptive values were provided as means (SD) or percentages of participants, and between-group comparisons were performed with general linear models (Proc GLM, SAS). For these analyses, total WAI was used as the dependent variable, and each of the muscular fitness tests was used as an independent variable (low, moderate and high corresponding to lower, medium and upper tercile for each respective sex). Both sexes were included in the analyses to favor the statistical power of the results. Analyses were controlled for age, sex and education. Results are reported as least square mean differences and 95% confidence intervals between the terciles.

## 3. Results

A total of 1005 PTs replied to the questionnaire. The majority of respondents were female (70.8%). Female participants had a mean age of 34.1 ± 7.9 (SD) years, and male participants had a mean age of 35.3 ± 8.5 (SD) years. The subgroup that performed physical fitness tests was composed of 68 participants, 29 women and 39 men. Table 1 shows the demographics of all participants as well as the subgroup performing the physical fitness tests.

Table 2 shows the WAI values stratified for age-group and sex (total as well as subscales) and muscular fitness values by sex. Overall, work ability was lower in those above 50 years of age, and this was slightly more evident in females. The most affected subscales were (1) current work ability compared with lifetime best (with the younger group as a reference, female aged 50 or older showed a difference of −0.3 points and male aged 50 showed a variation of −0.9 points), (2) work ability in relation to demands of the job (female and male aged 50 or older showed a difference of −0.5 points), (3) number of current diseases diagnosed by a physician (female aged 50 or older showed a difference of −0.6 points, and male aged 50 or older showed a variation of −0.8 points), and (6) own prognosis of work ability two years from now (with the younger group as a reference, female aged 50 or older showed a difference of −0.6 points and male aged 50 showed a difference of −0.3 points). The least affected subscale in all the age groups was (7) mental resources.

Table 3 shows the association between muscular fitness tests and work ability. The only significant association was that those participants with high performance in the Biering-Sørensen test scored 3.5 higher (95% CI) in WAI than those with low performance. No significant associations were observed between hand-grip strength or number of push-ups and work ability levels.

## 4. Discussion

The primary aim of the study was to describe the WAI values in Spanish PTs according to their age and sex. A difference of approximately −3 points in the overall WAI was observed both in men and women in the older age group compared to their younger counterparts. Furthermore, the overall WAI in women was nearly 2 points lower than in men in all age groups, in agreement with previous studies which have also described sex differences in favor of men among healthcare professionals [22]. However, on average, all the study groups remained in the category of good WAI (between 37 and 43 points), comparable to other occupational groups such as nurses [23].

Those subscales on the WAI that assessed subjective rather than objective aspects of work ability revealed greater age-related differences, including “current work ability compared with lifetime best”, where men and women aged ≥50 years showed differences of −0.8 and −0.6 points, respectively, compared to the younger groups and “work ability in relation to demands of the job”, where men and women aged ≥50 years showed differences of −0.9 and −0.3, respectively, compared to the younger groups. One possible explanation for this finding might be that while the demands of the job do not change, the physical fitness of the workers decreases with age. Therefore, the imbalance between physical workload and the physical fitness of the worker with advancing age could explain the poorer perception of work ability levels by older PTs in contrast to their younger counterparts. Supporting our results, a previous 11-year follow-up study reported a decline in the score of the index when ages ranged from 51 to 55 years, both in men and women in different occupations [8]. Although they did not include PTs in their sample, those participants with physically demanding jobs had a greater decline in their perception of work ability with aging. Other studies among healthcare workers have also found comparable results. For instance, a significant relationship was found between age and low perceived work ability among registered nurses from 10 different countries, suggesting that besides age itself, this association could be influenced by country-specific attitudes towards older workers, career opportunities, occupational health policies and the age at which pension can be drawn [9].

In our study, while sex differences were not excessively evident, the number of current diseases diagnosed by a physician was greater in females than in males. This could reveal the influence of other factors such as hormonal changes or physical disadvantages due to physical work demands, which may also be of explanatory importance. “Mental resources” was the only subscale that remained practically constant, showing no differences between sex or age groups.

Regardless, work ability is particularly difficult to discuss in absolutes, as it is the result of a complex interaction between the individual factors of the worker (including personal health, functional capacity, professional competence and values) and work-related factors (including working conditions, work demands, organization, management or leadership factors), as well as family support, the worker’s close social network and society in general [1]. It is beyond the scope of this study to analyze the associations of all these factors with work ability. However, most of the successful interventions that have been conducted to improve or prevent an early decline in work ability in physically demanding jobs have been focused on improving the physical capacity of the worker [24,25,26].

The second aim of this study was to investigate whether muscular fitness is associated with work ability among PTs. The main finding was that having higher performance in the Biering-Sørensen test was associated with better WAI scores (3.5 points higher) when compared to those who had a poorer performance. Even though hand-grip strength and push-up capacity could be considered as key factors for performing work-related tasks among PTs, the analysis did not reveal any significant association.

Those subjects who had better back-extensor muscle endurance (assessed with the Biering-Sørensen test) were found to have significantly higher scores in the WAI. This test is likely to be useful among PTs, as low back pain is the most frequently reported musculoskeletal complaint in this population [27]. The test has been shown to discriminate between subjects with low back pain and healthy controls and has also been shown capable of predicting the future onset of low back pain [28,29]. In healthy young subjects, the mean endurance time for this test has been shown to be 146 s in men and 189 s in women [30]. However, the mean back-extensor endurance time observed in PTs in this study (132.9 s among men and 92.8 s among women) is far below that observed by McGill. It could be argued that changes in back muscle endurance occur at a different rate in women than in men as they age; however, this result is somewhat surprising, as women have generally shown to have longer position-holding times than men [30,31] in healthy young populations. These results may be explained by the interaction of other factors such as previous back injury or current low back pain, which may account for the lower than expected scores. Moreover, the possible interference of psychological factors cannot be ruled out, as it has been suggested that besides muscle function or the intrinsic physiological fatigability of the trunk or hip muscles, psychological disturbances can also influence the performance of the Biering-Sørensen test [32]. Hence, it could conceivably be hypothesized that psychological factors such as depression, anxiety or psychological distress may be implicated in our results and could partly explain the low scores in the Biering-Sørensen test. However, since these variables have not been measured in the present investigation, future studies should explore the role of such factors in PTs’ physical performance. Interestingly, the association found between higher performance in this test and higher work ability levels suggests that back muscle endurance may play an important role in improving work ability levels or preventing an early decline in work ability among PTs, as it is required for many work-related tasks such as standing, lifting heavy objects or performing repeated movements. Future studies should target additional muscles involved in back stabilization which may provide further benefits to PTs.

Hand-grip strength is considered a predictor of future disability, morbidity and mortality, as well as a proxy measure of overall strength [33]. Moreover, an association between low hand-grip strength and low work ability has been previously reported, especially in workers exposed to hand–arm vibration [34]. Contrary to our expectations, the present study did not find any significant association between hand-grip strength and work ability. However, it is worth noting that the mean values of hand-grip strength in our study sample were below the mean score ranges for individuals of similar age and sex reported in the literature [35], where the mean handgrip strength among men with ages 25–29 and 30–34 years old has shown to be 49.7 and 46.5 kg, respectively, and that among women of the same age ranges has been shown to be 29.6 and 28.9 kg, respectively. One possible explanation for these findings might be that a maximal hand-grip is a task very unlikely to be frequently carried out by PTs, so the specific task-demands of this profession might have been underrepresented. Furthermore, although manual therapy is the primary type of treatment among Spanish PTs [11] and thus upper limb strength is deemed to be essential among PTs either for manual therapy or for lifting/transferring patients, several passive techniques exist. Further studies should be conducted to clarify the relationship between hand-grip strength and work ability levels among PTs, focusing on the specific task demands of the participants.

A previous systematic review reported that work-related pushing movements were significantly related to upper extremity symptoms, particularly for shoulder symptoms [36]. Because pushing is involved in many work-related tasks (i.e., handling or transferring patients or carrying heavy weights) and implies the activation of different upper body muscles, it could be assumed that a low pushing capacity could be associated with lower levels of work ability in PTs. According to previously established cut-off points in the healthy population [19], the average push-up capacity in our study sample can be considered “good” for men (“good” capacity ranges from 22 to 28 repetitions in younger subjects; in our study, male participants had a mean push-up capacity of 23.5 repetitions) and “normal” for women (“normal” capacity ranges from 10 to 14 repetitions in younger subjects; in our study, women had a mean push-up capacity of 10.4 repetitions). However, contrary to our expectations, this study failed to detect any consistent association between push-up capacity and WAI. It is plausible that pushing activities are not so frequently performed during a usual working day for PTs. Because this was not measured, the importance of this movement could have been overestimated. Furthermore, pain in the primary movers during a pushing action (i.e., anterior deltoid, pectoralis major and triceps brachii body) is not markedly prevalent among PTs, and thus these muscles might not be affected enough to influence WAI. This is especially relevant to understand why WAI is more sensitive to the underperformance of back muscle endurance tests, rather than to push-up capacity among PTs, as low-back pain is more prevalent and burdensome in this specific occupational group than shoulder pain. Moreover, not all PTs perform the same daily tasks, so those PTs who do not transfer patients or carry heavy weights during their working day will probably not need to have a high push-up capacity to have a good WAI. However, the effects of having higher overall strength cannot be ruled out, as push-up capacity has shown to be associated with a lower risk of cardiovascular disease events [37] and with lower cardiometabolic risk [38], which in turn could indirectly affect WAI.

During the last few decades, pension reforms have been carried out all over Europe, meaning that workers have to work more years in order to overcome the issue of labor shortages and a rise in government costs [39]. Some researchers have suggested that this could lead to a decline in the productivity level of older workers, as their muscular strength is expected to decrease due to the aging process [40], especially in those individuals engaged in physically demanding occupations, such as PTs. In the same manner, as muscular fitness declines over time, work ability has also been shown to be hampered as the workforce ages. Interventions among aging PTs should encourage the improvement of their muscle fitness levels to ensure that their work ability is maintained throughout their working life.

The most important limitation of the present study lies in its cross-sectional nature, as it cannot determine causality. Secondly, as the muscular fitness tests included in the study were not occupational task-specific tests to measure performance according to specific job demands, the PTs’ physical performance may have been underestimated. However, these tests were selected due to their validity, reliability, low cost and fast application. In addition, physical tests were performed by PTs from a convenience sample, which may limit their generalizability across the population. The healthy worker effect could have also been at play, as therapists who do not adopt preventive strategies may leave the profession earlier or change their job, and this is a possible explanation of the good work ability levels found in the older age group. However, the findings of the present study contribute to enhancing the current understanding of the characteristics of this profession and have important implications for developing further interventions among PTs. Importantly, this is the first attempt to associate objectively measured muscle fitness and WAI in PTs.

## 5. Conclusions

In the present study, WAI showed a decline in PTs with increasing age, in both males and females. Participants with higher back-extensor endurance scored significantly higher in WAI compared to those with low performance. Despite the cross-sectional nature of the present study, our results seem to highlight the importance of muscular fitness in PTs, especially that of back-extension endurance. Future studies are needed to confirm the potential role of muscular fitness in PTs to ensure that their work ability is maintained throughout their working life.

## Figures and Tables

**Table 1:** Demographics, lifestyle and education of women and men (all as well as the subgroup with physical fitness tests).

Variables	All		Physical Fitness Tests	
	Women	Men	Women	Men
n	712	293	29	39
Age (years)	34.1 (7.9)	35.3 (8.5)	36.1 (8.0)	39.5 (10.4)
Body Mass Index	22.5 (3.1)	25.2 (3.0)	22.8 (3.4)	25.2 (2.7)
Alcohol units per week	1.9 (2.1)	2.9 (2.7)	1.6 (2.1)	3.2 (3.0)
Moderate physical activity (min/week)	312.4 (459.5)	264.7 (397.7)	329.3 (555.2)	203.8 (266.8)
Vigorous physical activity (min/week)	85.7 (210.5)	69.2 (194.8)	133.3 (286.3)	33.2 (76.9)
Smoking				
No	88%	84%	79%	92%
Yes	12%	16%	21%	8%
Education				
Bachelor (3-year)	49%	48%	52%	49%
PhD	2%	2%	0%	3%
Bachelor (4-year)	25%	22%	28%	18%
Master	25%	28%	21%	31%

**Table 2:** Normative values of work ability (total score and subscales 1–7) as well as physical performance.

WAI and Physical Performance	Women			Men		
	<30 Years	30–49 Years	≥50 Years	<0 Years	30–49 Years	≥50 Years
n	239	441	32	78	194	21
WAI, total score	41.0 (4.4)	40.4 (4.9)	38.5 (7.8)	43.1 (3.8)	42.0 (4.8)	40.0 (6.3)
WAI_1, current work ability compared with lifetime best	8.4 (1.5)	8.4 (1.3)	8.1 (1.8)	8.7 (1.4)	8.7 (1.3)	7.8 (1.9)
WAI_2, work ability in relation to demands of the job	8.4 (1.2)	8.2 (1.1)	7.9 (1.2)	8.7 (1.2)	8.5 (1.3)	8.2 (1.6)
WAI_3, number of current diseases diagnosed by a physician	3.8 (1.8)	3.6 (2.0)	3.2 (2.4)	4.9 (1.9)	4.6 (1.8)	4.1 (1.7)
WAI_4, estimated work impairment due to diseases	5.3 (1.0)	5.2 (1.0)	5.1 (1.2)	5.4 (1.0)	5.4 (0.8)	5.0 (1.3)
WAI_5, sick leave during the past year	4.6 (0.9)	4.5 (0.9)	4.5 (1.0)	4.8 (0.4)	4.5 (0.9)	4.7 (0.9)
WAI_6, own prognosis of work ability two years from now	6.9 (0.6)	6.8 (0.9)	6.3 (1.7)	6.9 (0.5)	6.7 (1.0)	6.6 (1.4)
WAI_7, mental resources	3.6 (0.6)	3.6 (0.6)	3.7 (0.6)	3.7 (0.6)	3.7 (0.6)	3.7 (0.5)
n	29			39		
Handgrip strength (kg)	26.9 (4.9)			43.6 (6.7)		
Push-ups (number)	10.4 (5.6)			23.5 (10.3)		
Biering-Sørensen (s)	92.8 (42.6)			132.9 (59.4)		

Abbreviations: WAI, Work Ability Index.

**Table 3:** Least square mean differences (95% CI) of work ability (WAI) among people with low, moderate and high physical performance in handgrip, push-ups and the Biering-Sørensen test (low, moderate and high corresponding to lower, medium and upper tercile for each respective sex). Analyses are controlled for age, sex and education.

Performance	Handgrip Strength (kg)	Push-Ups (Number)	Biering-Sørensen (s)
Moderate-Low	−1.1 (2.2 to −4.5)	−1.3 (2.1 to −4.7)	0.7 (3.9 to −2.6)
High-Low	0.6 (4.0 to −2.7)	1.3 (4.7 to −2.0)	**3.5 (6.8** to **0.2)**
High-Moderate	1.8 (5.1 to −1.5)	2.6 (5.9 to −0.6)	2.8 (6.1 to −0.5)

Boldface type indicates statistical significance.
